# Identification of conserved transcriptome features between humans and *Drosophila* in the aging brain utilizing machine learning on combined data from the NIH Sequence Read Archive

**DOI:** 10.1371/journal.pone.0255085

**Published:** 2021-08-11

**Authors:** Joe L. Webb, Simon M. Moe, Andrew K. Bolstad, Elizabeth M. McNeill

**Affiliations:** 1 Department of Food Science and Human Nutrition, Iowa State University, Ames, IA, United States of America; 2 Department of Electrical and Computer Engineering, Iowa State University, Ames, IA, United States of America; 3 Neuroscience Interdepartmental Graduate program, Iowa State University, Ames, IA, United States of America; Soongsil University, REPUBLIC OF KOREA

## Abstract

Aging is universal, yet characterizing the molecular changes that occur in aging which lead to an increased risk for neurological disease remains a challenging problem. Aging affects the prefrontal cortex (PFC), which governs executive function, learning, and memory. Previous sequencing studies have demonstrated that aging alters gene expression in the PFC, however the extent to which these changes are conserved across species and are meaningful in neurodegeneration is unknown. Identifying conserved, age-related genetic and morphological changes in the brain allows application of the wealth of tools available to study underlying mechanisms in model organisms such as *Drosophila melanogaster*. RNA sequencing data from human PFC and fly heads were analyzed to determine conserved transcriptome signatures of age. Our analysis revealed that expression of 50 conserved genes can accurately determine age in *Drosophila* (*R*^2^ = 0.85) and humans (*R*^2^ = 0.46). These transcriptome signatures were also able to classify *Drosophila* into three age groups with a mean accuracy of 88% and classify human samples with a mean accuracy of 69%. Overall, this work identifies 50 highly conserved aging-associated genetic changes in the brain that can be further studied in model organisms and demonstrates a novel approach to uncovering genetic changes conserved across species from multi-study public databases.

## Introduction

Employing a comparative approach to study conserved aging phenotypes across multiple species provides a deeper insight into molecular aging signatures than studying aging within a single organism. Since aging is the strongest risk factor for developing neurodegenerative diseases, identification of conserved molecular signatures in the brain will allow for more effective study of mechanisms that may underlie neurodegeneration as well as facilitate the development of novel therapeutic strategies to slow cognitive aging [1].

Age-related cognitive decline varies across individuals. Neuroimaging studies indicate that cognitive decline is related to changes in the structure of the prefrontal cortex (PFC) [1] and altered white matter integrity [2]. The PFC is vulnerable to age-related morphology changes [3], with age-related alterations in the cortex occurring across multiple species [2]. Neuronal transcription plays a key role in regulating cognitive function and neural transmission [4], but the extent to which gene expression in the PFC is conserved across species in aging is relatively unknown. A subset of age-related transcriptome changes in the PFC are known to be conserved between humans and mice [5]. In *Drosophila*, study of neurodegenerative diseases frequently utilizes both the brain, containing the mushroom body which is necessary for olfactory learning and memory [6], and the complex eye of adult *Drosophila* [7]. Identifying conserved age-related transcriptome changes across model organisms allows for more efficient mechanistic study and may uncover novel therapeutic pathways for preventing age-related cognitive decline or vulnerability to neurodegeneration.

Here, we demonstrate the utility of previously published, publicly available RNA sequencing datasets from humans and *Drosophila* to increase statistical power through combining samples across multiple laboratories. This facilitates the identification of conserved aging genes in the human PFC/fly head in a unique approach that circumvents the prohibitive cost of collecting hundreds of cross-species samples in a single laboratory. In this work, we examine transcriptome changes across species to identify conserved genes that are highly correlated with chronological age in both human and fly. Furthermore, we were able to predict genetic network interactions of these genes as well as pathways affected by the subset of conserved genes. From a comparative biology perspective, such conserved genes should play an important role in age-related physiological changes in the PFC and comprise a promising target set for future mechanistic analysis in *Drosophila*, a model organism with a short lifespan and well-established genetic tools. We identify the target gene set by cross-referencing genes highly correlated with age in humans and *Drosophila*. Machine learning algorithms validate the association of these genes with age. Similar techniques could be used to identify target orthologs in other model organisms and tissues in future analyses.

## Materials and methods

### Data selection and acquisition

Available sequencing data was identified using the terms “aging”, “RNA-seq”, “brain transcriptome”, “cortex”, and “heads” in articles on PubMed and in the NIH Sequence Read Archive (SRA) database. All eligible studies were published online before August 2019. The following inclusion criteria were used to select data for the analysis: (1) tissue (fly head, human prefrontal cortex); (3) availability of age data; (2) lack of disease (e.g., traumatic brain injury) or treatment; and (4) Illumina format raw next-generation sequencing files. The following exclusion criteria were also used to select data for this study: (1) lack of neurologically normal samples; (3) incomplete age information or incubation temperature data for fly samples; (2) presence of a treatment such as injections, traumatic brain injury or sham-surgery procedures; and (4) studies including only microarray data. When overlapping data of the same study population was encountered in more than one publication across multiple SRA submissions, only the most complete study was used for our analysis leaving a single occurrence for each biological sample. To compare orthologous genes across species, the *Drosophila* RNAi Screening Center Integrative Ortholog Prediction Tool (DIOPT; http://www.flyrnai.org/diopt) [8], was used to identify orthologs between humans and *Drosophila*. Publicly available data downloaded from the NIH SRA included multiple Bioprojects outlined in **Table 1**. Files were downloaded from the SRA using Aspera Connect and reads that were downloaded in SRA format were converted using fastq-dump from the NIH SRA-Toolkit [9]. Data were subdivided into three groups for each species. Young was <30 years in humans and ≤10 days in *Drosophila*, Middle-aged was between 30–60 years in humans and between 10–29 days in *Drosophila*, Old was classified as above 60 years in humans and above 30 days in *Drosophila*. Additional sequencing parameters such as read lengths or study metadata can be found for each study contained within this analysis in the NIH Sequence Read Archive (SRA) according to the Bioproject identifiers within **Table 1**.

**Table 1:** Study Characteristics: In this table, all publicly available data that were aggregated for this study are described, along with their Sequence Read Archive bioproject numbers, sample descriptors and average number (#) of reads.

Bioprojects	Species	Sample Size	Age Groups	Tissue Type	Citation	Average # of reads
PRJEB7674	Human	10	Y/M/O	Prefrontal Cortex	[10]	31M
PRJNA322318	Human	21	Y/M/O	Prefrontal Cortex	[11]	71M
PRJNA394722	Human	19	Y/M	Prefrontal Cortex	[12]	31M
PRJNA398545	Human	4	Y	Prefrontal Cortex	[13]	56M
PRJNA213747	Human	12	Y	Prefrontal Cortex	[14]	7.3M
PRJNA222268	Human	15	Y/M/O	Prefrontal Cortex	[15]	9.5M
PRJNA271929	Human	35	M/O	Prefrontal Cortex	[16–18]	43M
PRJNA505319	Fly	27	Y/M/O	Head	[19]	2.7M
PRJNA388952	Fly	21	Y	Head	[20, 21]	9M
PRJNA270175	Fly	32	M/O	Head	[22]	19M
PRJNA379297	Fly	6	O	Head	[23]	2.4M
PRJNA432934	Fly	10	M	Head	[24]	15M
PRJNA320747	Fly	12	O	Head	[25]	34M

Age groups are abbreviated young (Y), middle (M) and old (O).

### Quality control & adapter trimming

All reads were analyzed with FastQC for quality control. Reads with low quality scores (average quality < 10) were discarded. Adapter sequences were trimmed using BBDUK [26]. Reads that matched known Truseq or Nextera adapter sequences were removed during trimming. Individual study manuscripts and supplemental data were examined to identify if reads were sequenced using a forward or reverse library preparation kit.

### Alignment & read quantification

Reference fasta genome files and genome annotation gtf files were downloaded from the Ensemble genome browser and Flybase.org browser. Human reads were aligned to the GRCh38 release 94 of the Ensemble Human Genome, and fly reads were aligned to the DMEL release 25 Flybase genome using the STAR v2.5.2 aligner [27]. Transcripts aligning to specific genes were counted using STAR with the quantMode geneCounts function to map transcripts to each genome. Files containing gene counts for all samples are available on GitHub at: https://github.com/akbee/Aging-Brain-Transcriptome.

### Algorithm selection

Based on previously published work [28, 29], 13 algorithms were selected for regression and classification. The top performing algorithms using default hyperparameters were then used for all subsequent analysis. All models were assessed using all available transcriptome data.

### Regression & classification

To identify correlation with biological age, all data were split into a training set (75%) and a testing set (25%) by randomly selecting samples using the train_test_split module in Scikit-learn. Age association models were fit using Scikit-learn v0.23.2 [30] in Jupyter notebooks v.6.1.4 running Python 3.7.3. XGBoost, Gradient Boosting, ADABoost, Bagging Regressor, Random Forest, Extra Trees Regressor, K-nearest neighbors (KNN), Logistic Regression, Linear Discriminant Analysis (LDA), Naive Bayes, Linear Regression, Huber Regression, and ARD Regression were implemented with their default settings in Scikit-learn. Regression performance was evaluated based on mean absolute error, *R*^2^, and median absolute error scores in Scikit-learn. All analyses were conducted in batches of 1000 random sampling tests with replacement to estimate the mean for each model performance metric and a 95% confidence interval for *R*^2^. In-depth descriptions of these algorithms can be found in Hastie, et al. [31].

### Data normalization approaches

We examined Trimmed Mean of M values (TMM) [32], and the more common Relative Log Expression (RLE) method [33] for RNA sequencing read counts. We found that TMM normalization provided more robust regression and classification results. Histograms of log read counts also suggest that TMM normalization removes study effects better than RLE normalization (see **S1 Fig**).

### Calculation of conserved genes

All genes within species were ranked according to correlation coefficient according to within-species age shown in **S1 Table**. The top 1000 human aging correlated genes were converted into fly homologs using Diopt version 8.0. Human fly homologs were prioritized according to highest to lowest score within both human ensembl ID and flybase ID and the highest ranked homolog for each gene was kept. This left a one to one ‘best match’ between human ensembl ID and flybase ID. This list was cross-referenced to the top 1000 fly aging correlated genes to find matches between the two lists.

### Heatmap construction

Expression data of human prefrontal cortex at young, middle, and old was input to the Next-Generation Clustered Heat map (NG-CHM) version 2.16.0 [34].

### Genetic interactome analysis with cytoscape

Cytoscape version 3.8.0 was used to generate a genetic interactome of 50 human ensembl IDs with a medium confidence score [35]. STRING (Search Tool for the Retrieval of Interacting Genes/Proteins)—Protein Query generated a node network that allowed functional enrichment data of all 50 genes to be generated. The Kyoto Encyclopedia of Genes and Genomes (KEGG) database was queried to identify the functional pathways corresponding to each gene in each species. We report those pathways with a false discovery rate (FDR) corrected significance of p ≤ 0.005. Aging correlated genes were cross-referenced to STRING–disease database [36] using a threshold of 1000 genes annotated as disease associated.

### Phylogenetic tree mapping

Cyverse DNA Subway was utilized to map all 50 conserved human genes to 49 *Drosophila* genes. URL: www.cyverse.org [37]. Protein sequence similarity of all conserved genes was included. Bootstrapped scores of 100 trials are indicated as numerical representations connecting genes to each other.

### Panther analysis

Panther (Protein ANalysis THrough Evolutionary Relationships) Classification System (version 15) was used to categorize molecular and cellular classification of genes of interest [38] (http://www.pantherdb.org). Gene Ontology (GO) Analysis was used to group genes according to cellular location, function and biological processes.

### Data and software availability

The data used in this study are publicly accessible through the National Institutes of Health Sequence Read Archive (SRA) [39]. All Ascension numbers can be found in the supplementary data files, along with BioProject numbers in **Table 1**. The Jupyter notebook describing the workflow, as well as other scripts to conduct analyses within this manuscript are publicly available on GitHub at: https://github.com/akbee/Aging-Brain-Transcriptome.

### Ethics approval and consent to participate

All studies contained within this manuscript received ethics approval from their respective IRB ethics committees prior to study initiation. See citations in table one for additional information on each published study. Iowa State University Institutional Review Board does not require approval for the secondary data analysis of anonymous publicly available data used in this work.

## Results

### Combination of publicly available transcriptome datasets

To obtain a higher analytical power than typically feasible from a single experiment in human prefrontal cortex and *Drosophila* head, we explored methods and constraints for combining data from the National Institutes of Health Sequence Read Archive. We identified key inclusion and exclusion requirements for the combination of data from this publicly available repository. These factors are described in detail in the methods section. The studies meeting these criteria and used for further analysis are described in **Table 1**. In total, 289 raw fastq files were downloaded from NCBI and processed. Ninety-four percent of the *Drosophila* reads and ninety-six percent of the human reads were mapped to their respective reference sequences. In our analysis of the combined data sets, 9,650 genes in *Drosophila* and 16,879 genes in humans had read values of greater than 10 in at least 50% of the samples.

### Predictive algorithms for chronological age regression and classification

For regression analysis, we compared 13 distinct algorithms in the Python Scikit-learn library for predicting human chronological age as shown in **Table 2A and 2B.**

**Table 2:** Algorithm evaluation.

Algorithm	Mean *R*^2^	Mean square error	Median absolute error	*R*^2^ (95% CI)
**A. Algorithm Evaluation—Human**				
XGBoost	0.62	217.05	8.28	34.6%-80.5%
Gradient Boosting	0.61	218.12	8.72	36.71–75.52
Adaboost	0.58	235.78	9.27	24.43–78.04
Bagging Regressor	0.52	269.16	10.56	52.31–52.32
Random Forest	0.52	269.70	10.68	52.01–52.02
Extra Trees Regressor	0.48	290.43	10.75	47.84–48.89
KNN	0.38	341.94	14.10	22.48–60.16
Logistic Regression	0.21	440.48	11.06	0.0–64.73
LDA	0.13	481.82	12.37	13.4–21.84
Naive Bayes	-0.08	605.21	15.15	.-8.94–7.3
Linear Regression	-0.43	876.33	16.95	.-12.74–2.4
Huber Regression	-1.01	1115.84	18.40	-2.87
ARD Regression	-2.75	2084.02	16.16	-22.95
**B. Algorithm Evaluation—*Drosophila***				
XGBoost	0.89	29.23	0.40	71.0–99.0
Gradient Boosting	0.89	29.03	0.49	63.3–99.3
Adaboost	0.94	14.72	0.59	82.0–99.5
Bagging Regressor	0.86	34.85	1.14	60.1–98.1
Random Forest	0.87	34.15	1.14	63.0–98.0
Extra Trees Regressor	0.94	13.82	0.48	86.0–99.2
KNN	0.66	88.50	2.92	31–86.9
Logistic Regression	0.88	29.01	0.00	57.2–100
LDA	0.77	57.10	0.00	26.0–97.9
Naive Bayes	0.64	89.64	0.01	1.4–93.5
Linear Regression	-0.61	411.27	5.06	0.0–70.4
Huber Regression	-0.21	310.11	7.03	0.0–45.5
ARD Regression	0.07	235.55	6.01	0.0–77.8

In this table, algorithms were evaluated according to their ability to predict chronological age using gene expression data from all available genes. Rows indicate the results for an individual regressor across 1000 bootstrapped random draws training on 75% of the samples and testing on 25%. A) Human algorithm selection. B). *Drosophila* algorithm selection. All values are reported as the mean across 1000 iterations.

XGBoost performed best with a mean *R*^2^ of 0.62 and a median absolute error of 8.28 years on held out data. The standard gradient boosting regressor through Scikit-learn performed similarly with a mean *R*^2^ of 0.61 and a median absolute error of 8.72 years **(Table 2A)**. Within *Drosophila*, XGBoost performed among the best algorithms, with a mean R^2^ of 0.89. Linear regression, which has been shown previously to predict age from the transcriptome in peripheral blood [40], performed poorly on our data set of complex heterogeneous tissue samples **(Table 2A and 2B)**. Additional details of human and *Drosophila* regression results using XGBoost can be found in **S2 Table**. Given its superior predictive power on this specific data set, XGBoost was selected for downstream analysis.

Comparison of algorithms within *Drosophila* resulted in several algorithms with similar predictive abilities. Four algorithms had a mean *R*^2^ value of 0.89 or greater. Of these, XGBoost had the lowest median absolute error. Due to its strong predictive ability in both human and *Drosophila* in comparison to our other algorithms, XGBoost was selected for downstream analysis.

Algorithms were also compared for classifying samples. This approach is valuable in data sets where exact age is unknown. In a previous report examining machine learning to classify human age groups, an ensemble classifier using linear discriminant analysis (LDA) most accurately classified human chronological age [41]. In our analysis of human PFC and whole fly head, we found that LDA resulted in a mean accuracy of .85 for fly and .73 for human data, respectively. XGBoost performed better with a mean accuracy of .93 for fly and .80 for human data, respectively (**S3 Table**). Overall, classification of both human and *Drosophila* samples was possible with a high degree of accuracy **(S2 Fig)**.

### Identification of conserved genomic predictors of chronological age in human and *Drosophila*

**Fig 1:**
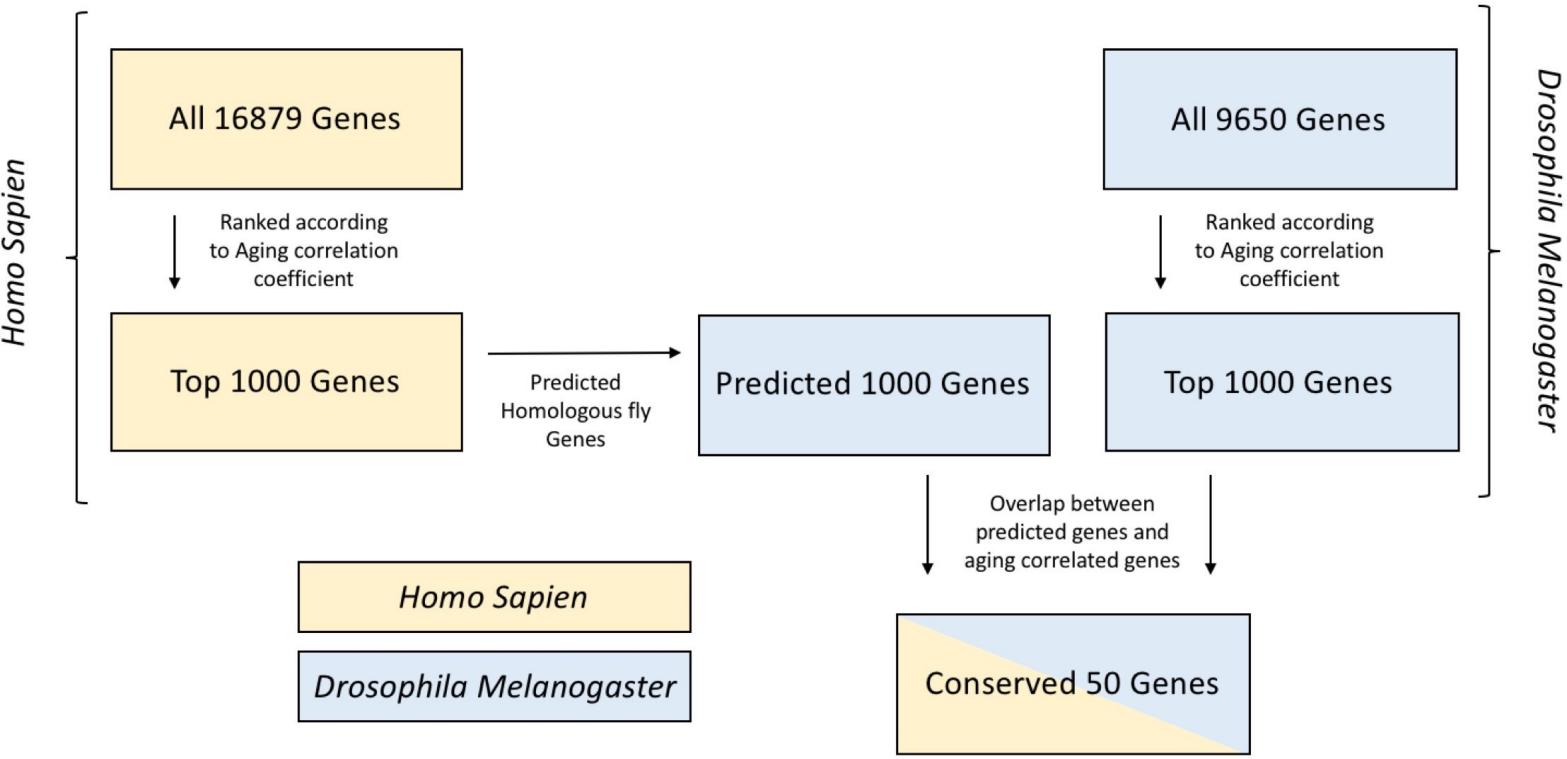
Workflow of biological age prediction using XGBoost across species. Depicting selection of genes for both aging involvement and conservation between Human and *Drosophila*.

**Fig 2:**
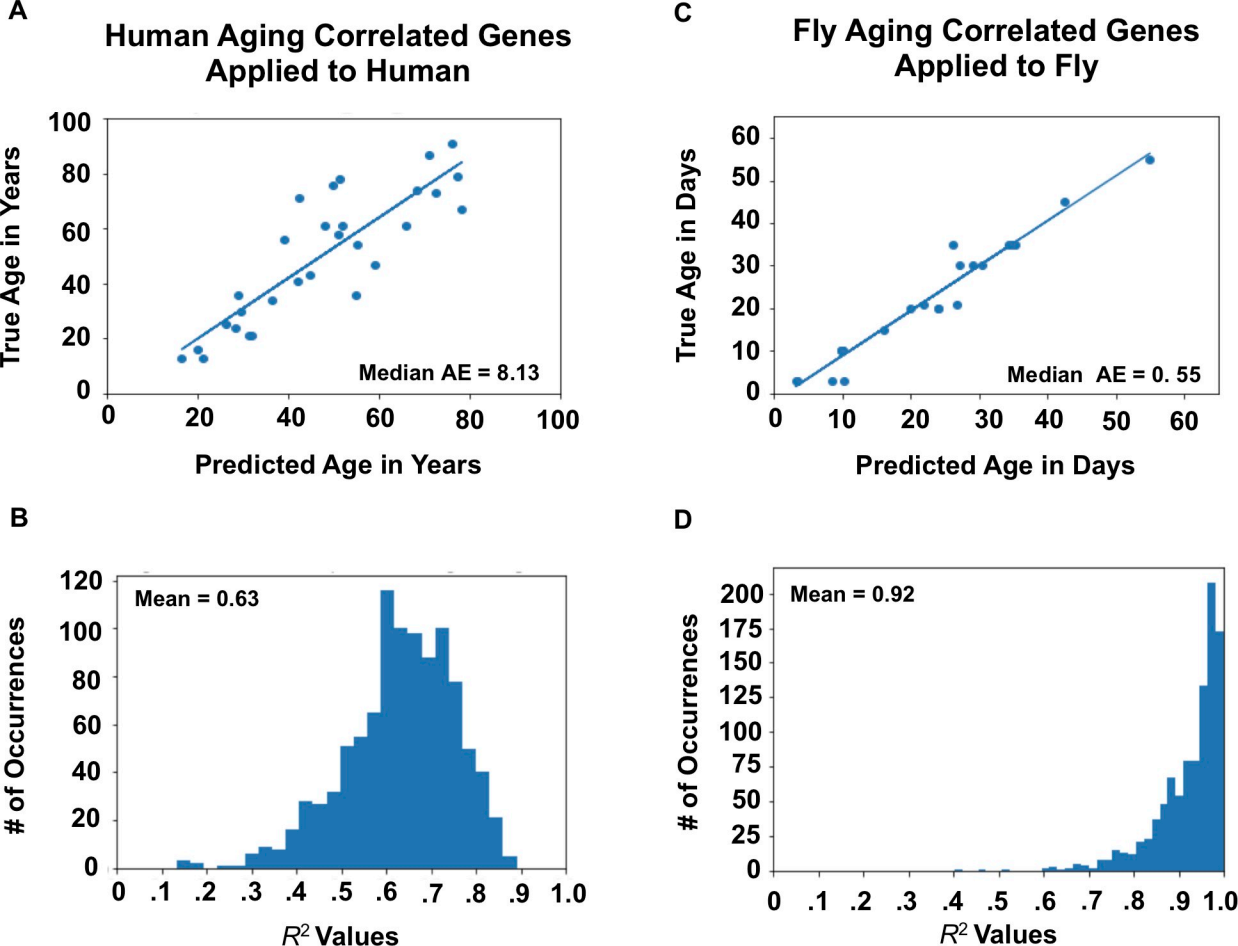
Biological age prediction using XGBoost across species. A) Sample regression analysis using all human genes to predict human age. B) Histogram of *R*^2^ values for predicting human age with all available human genes with a mean R^2^ of 0.61. C) A sample regression analysis with all *Drosophila* genes to predict *Drosophila* age. D) Histogram of R^2^ values for predicting *Drosophila* age using all available *Drosophila* genes with a mean R^2^ of 0.93. All results calculated using XGBoost. Histograms represent averages across 1000 bootstrapped random samplings where the regressor or classifier was trained on 75% of the samples and tested on 25% with values reported as the mean across 1000 iterations. Median AE stands for Median Average Error.

**Fig 3:**
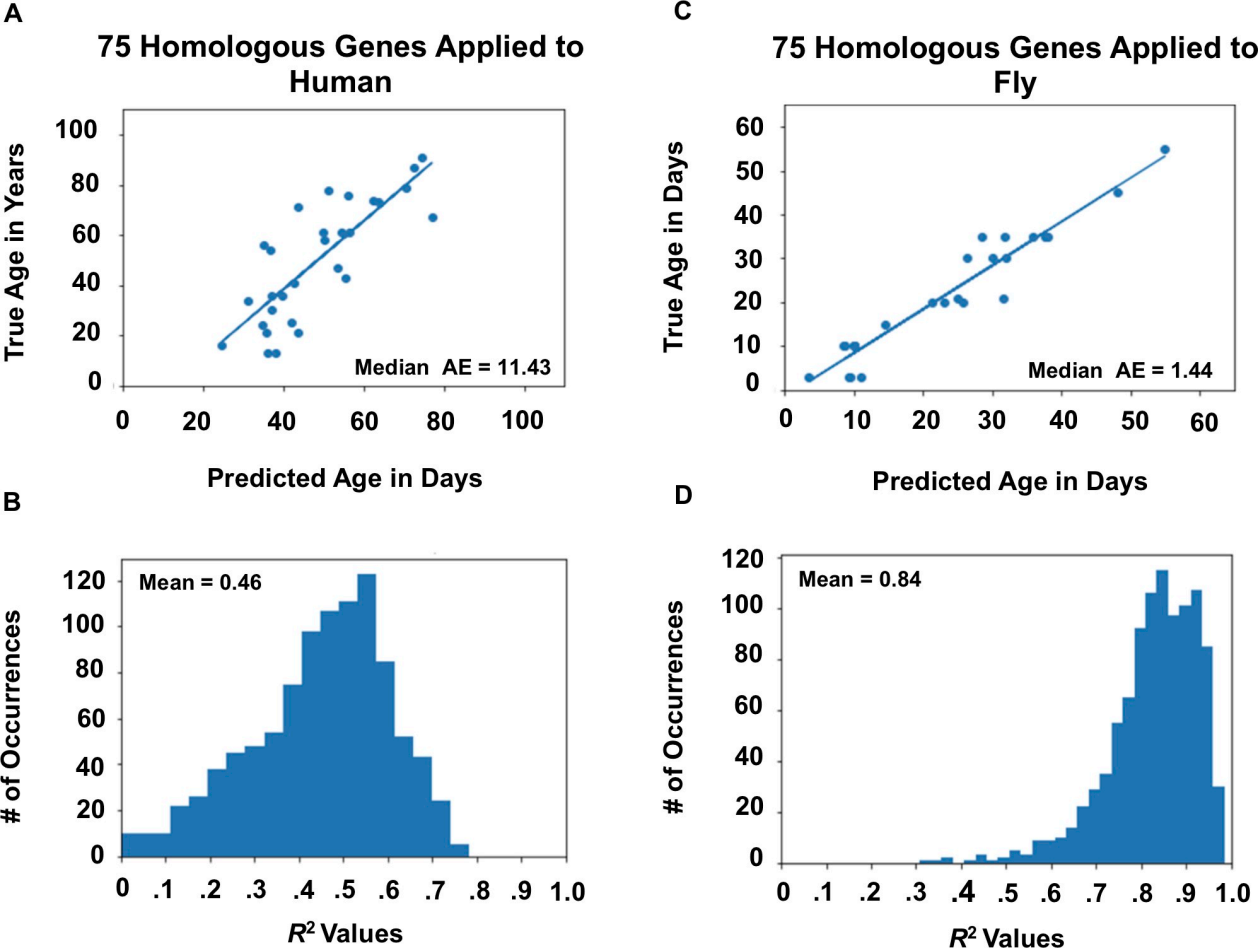
Feature selection using aging correlated genes across species. A) Sample regression analysis using the top 1000 human aging correlated genes to predict human age. B) Histogram of R^2^ values using the top 1000 human aging correlated genes to predict human age with a mean R^2^ of 0.62. C) Sample regression analysis predicting *Drosophila* age using the top 1000 aging correlated *Drosophila* genes. D) Histogram of R^2^ values predicting *Drosophila* age using the top 1000 *Drosophila* aging correlated genes with mean R^2^ of 0.95. All results calculated using XGBoost. All histograms represent averages across 1000 bootstrapped random samplings where the regressor or classifier was trained on 75% of the samples and tested on 25%. All histogram values are reported as the mean across 1000 iterations. Median AE stands for median average error.

A workflow depicting our approach for conserved gene identification can be found in **Fig 1**. First, we established that gene expression data in the human PFC and fly head were predictive of chronological age within species. **Fig 2A** shows a sample regression result for human data. These results were obtained using 75% of the samples to train and the remaining 25% for testing. To eliminate the chance of a “lucky draw,” we repeated the 75/25 split 1000 times to assess average performance. **Fig 2B** depicts the resulting histogram of *R*^2^ values. The mean *R*^2^ was 0.61, and the median absolute error was 8.07 years. **Fig 2C and 2D** shows results for fly data, where a mean *R*^2^ of 0.93 and a median absolute error of 0.46 days were obtained. The average accuracy of classifying samples as young, middle-aged, or old was 80% for humans and 93% for flies (**S3 Table**). We repeated this procedure using the 1000 genes most correlated with aging in humans and the 1000 genes most correlated with aging in flies. Using this technique to reduce the number of features in our models resulted in a slight boost in *R*^2^ on the testing data for both species, illustrating the potential for error due to overfitting in machine learning models (Compare **Figs 2** and **3**).

**Fig 4:**
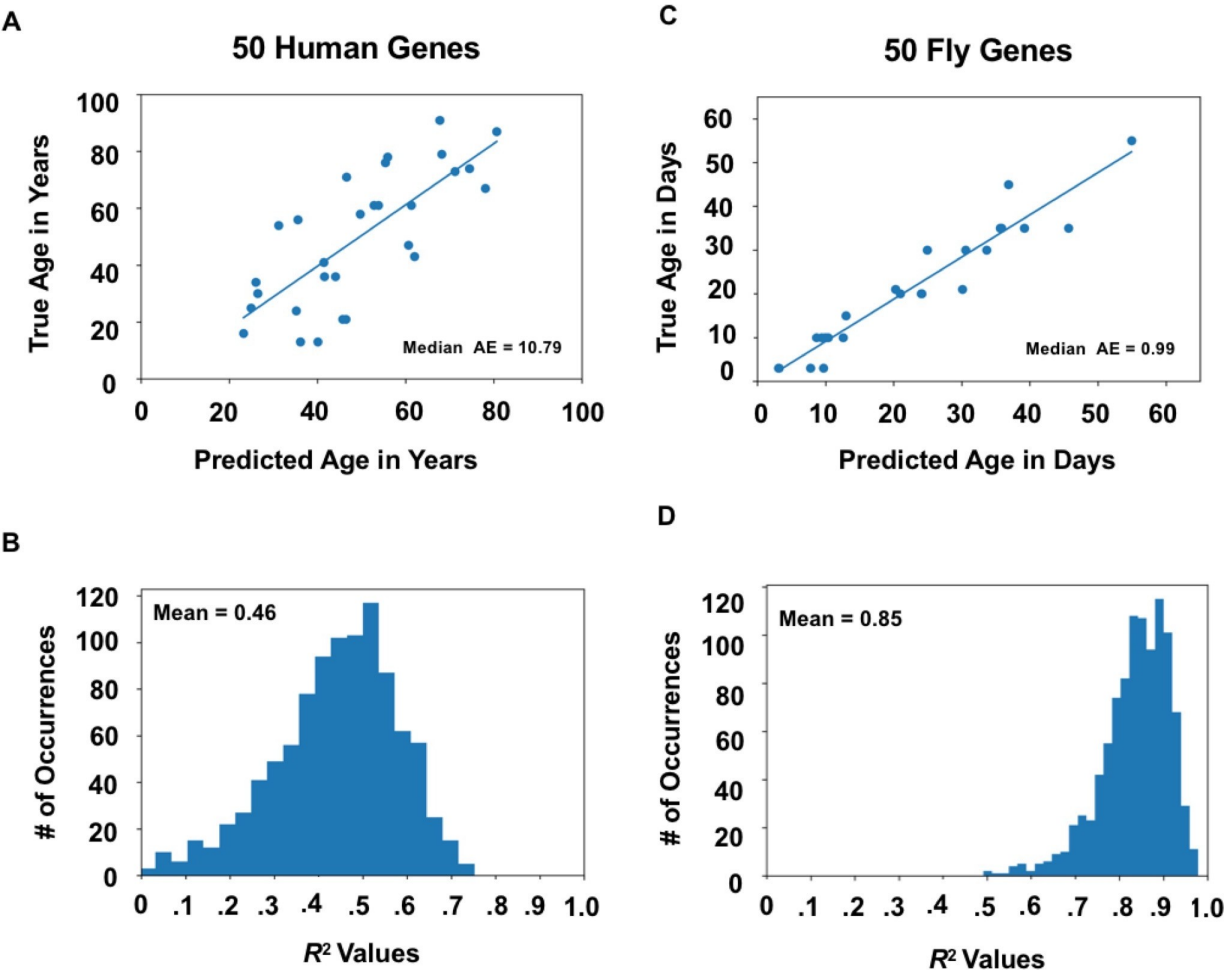
Intersection of homologous aging-correlated genes. A) Sample regression analysis of the overlapping 50 aging correlated genes across humans. B) Histogram of R^2^ values of human age prediction using the conserved 50 aging correlated genes with a mean R^2^ of 0.46. C) Sample regression analysis of the mean R^2^ values predicting *Drosophila* age using the 50 aging correlated genes. D) Histogram of R^2^ values predicting *Drosophila* age using the conserved 50 aging correlated genes with a mean R^2^ of 0.85. All results calculated using XGBoost. All histogram results represent averages across 1000 bootstrapped random samplings where the regressor or classifier was trained on 75% of the samples and tested on 25%. All histogram values are reported as the mean across 1000 iterations. Median AE stands for Median Average Error.

After determining that aging correlated genes retain predictive ability within species, we wanted to identify a smaller set of genes that could be validated in future animal studies examining their role in aging. Cross referencing the 1000 genes most important in aging within both human and *Drosophila* through Diopt, we identified genes shared among both lists. This narrowed our window of conserved genes to 50 which were highly predictive of aging in both humans and flies. **Fig 4** illustrates the ability to identify human and *Drosophila* age based on expression levels of these 50 genes. **Fig 4A and 4B** depicts the human results, where these 50 genes identified chronological age with a mean *R*^2^ of 0.46 and median absolute error of 10.79 years. **Fig 4C and 4D** depicts the results for *Drosophila*, where a mean *R*^2^ = 0.85 and median absolute error of 0.99 days was obtained. In both cases, we pay a small penalty in regression accuracy for reducing the number of genes from 1000 to 50, however both predictions remain strong (see **S2 Table**). Similar results hold for classification accuracy (see **S3 Table**). For example, reducing the number of genes used in the prediction by 95% results in only a 15% drop in human age classification accuracy and a 10% drop in fly classification accuracy.

### Correlated genes enriched in aging signaling pathways

**Fig 5:**
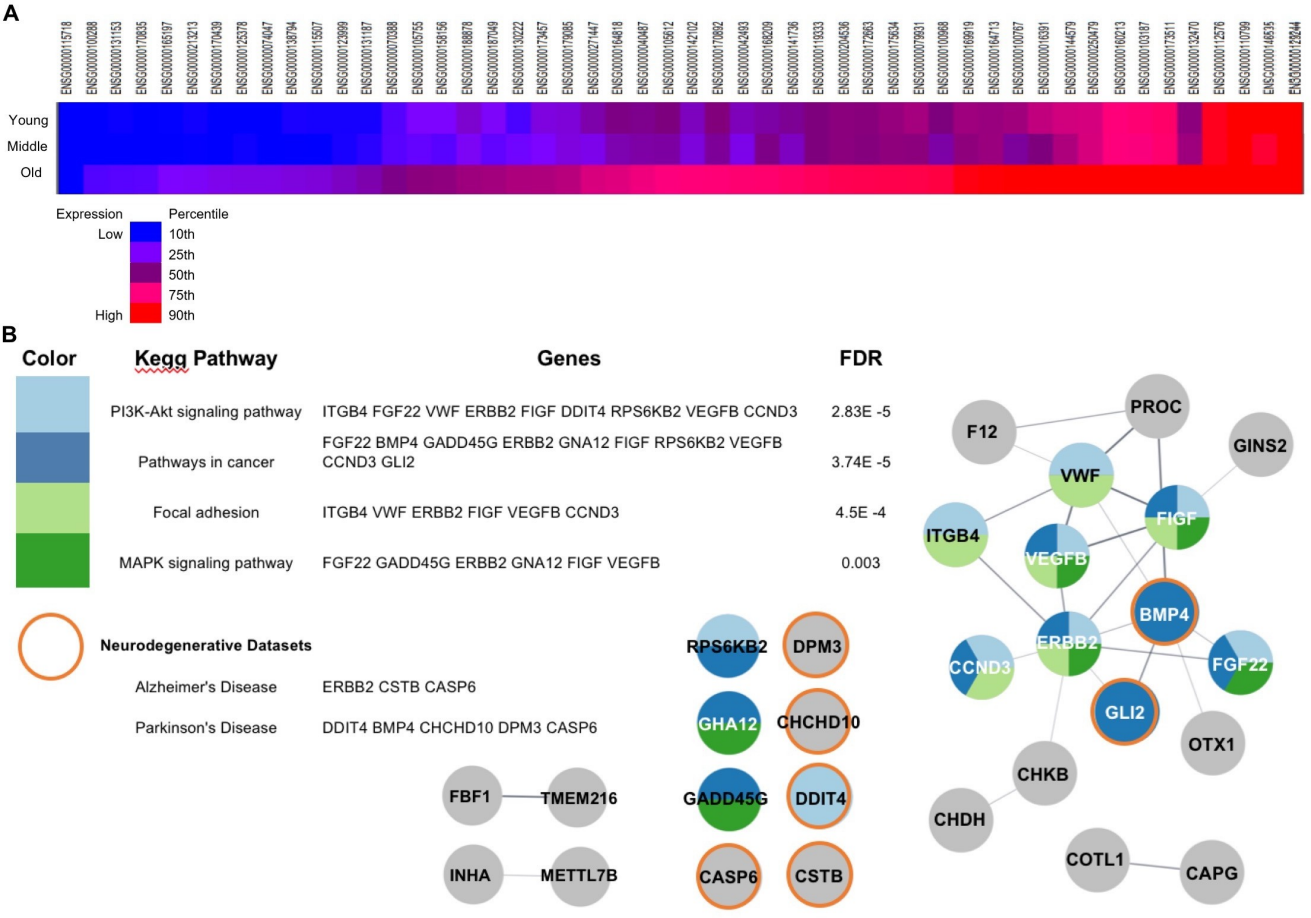
A) A heat map of gene expression in young, middle aged, and old human prefrontal cortex of the 50 conserved genes. Shift of color from blue to red indicates an increase in expression relative to all gene expression in the dataset as indicated B) Interactome representing STRING Network interactions of 50 conserved genes. Networks connected through ‘edge’ lines represented by STRING confidence score. Line thickness indicates interaction confidence scores with greater thickness indicating higher confidence interaction. The table on the right indicates KEGG pathways represented by the STRING network. Colors in nodes represent involvement in pathway, see table. Orange border surrounding a node indicates occurrence within 1000 genes with a reported association of either Parkinson’s or Alzheimer’s disease.

A heatmap of mean human expression data from the prefrontal cortex vs. age in the 50 highly conserved genes indicates an overall increase in expression from young to old age groups in the 50 highly correlated conserved genes (**Fig 5A**). Higher expression is indicated in red with lower expression indicated in blue.

Genetic interactions and enrichment of genes in pathways previously known to play an important role in brain aging are revealed using STRING analysis of the 50 highly conserved correlated genes. **Fig 5B** represents a genetic interactome in which genes are connected based on a STRING confidence score reflecting functional associations. Functional enrichment with KEGG pathways was referenced to identify pathways associated with the 50 genes. Two key pathways known to play an important role in aging were revealed in this analysis. These included the PI3K-akt (9 genes) and MAPK (6 genes). The enrichment of these signaling pathways was found to be significant and survived FDR multiple testing correction (p ≤ 0.005). We observed a high amount of overlap with the genes represented in these two pathways and other significantly enriched pathways such as cancer and focal adhesion as is indicated by the highly connected group of genes which are found in each of these pathways **Fig 5B (color coded circles)**.

As age is one of the greatest predictors of neurodegeneration, our list of 50 genes identified from PFC and fly head were referenced to a STRING disease database. 1000 Genes with a reported association with either Parkinson’s or Alzheimer’s disease were cross-referenced to the list of 50 conserved genes. Within the 1000 genes associated with Alzheimer’s disease and Parkinson’s disease, a total of 7 genes were also identified in our conserved list. The genes associated with Alzheimer’s disease were Erb-B2 receptor tyrosine kinase 2 (ERBB2), Cystatin B (CSTB), and Caspase 6 (CASP6). Those associated with Parkinson’s disease were DNA damage inducible transcript 4(DDIT4), Bone morphogenic protein 4 (BMP4), Coiled-coil-helix-coiled-coil-helix domain containing 10 (CHCHD10), Dilochyl-phosphate mannosphyltransferase 3 (DPM3) and caspase 6 (CASP6) (See orange outline in **Fig 5B**).

We utilized the Panther Gene Ontology database to reveal evolutionarily relevant functions of our aging correlated genes. Panther Gene Ontology utilizes phylogenetic information to infer evolutionary function of genes that have yet to be well characterized. Genes highly correlated with aging were mapped to Panther pathways as shown in **S3A Fig**. Panther analysis of the top 1000 human aging-correlated genes indicated that the most highly represented genetic pathway was Gonadotropin-releasing hormone (P06664) with 10 genes represented. Reducing our input to the 50 conserved human genes, 4 remained in the Gonadotropin pathway **(S3B Fig)**. Although the Gonadotrophin pathway is emerging as a possible therapeutic target in aging and neurodegeneration in Humans [42], The gonadotropin-releasing hormone pathway has not been well characterized in *Drosophila*.

To elucidate roles of the conserved Gonadotropin-hormone genes in *Drosophila*, we constructed a phylogenetic tree using our 50 conserved genes of Human and *Drosophila*. Protein sequences of our conserved genes were input into Cyverse–DNA Subway to generate a full phylogenetic tree. **S3C Fig** depicts a sub-cluster of genes from our phylogenetic tree. Interestingly, 2 of our genes from the Gonadotropin-releasing hormone pathway appeared to share sequence similarity, including ENSG00000123999 (INHA) and ENSG00000125378 (BMP4), along with its homolog FBgn0000490 (dpp).

## Discussion

In this work, we developed a method for combining datasets across multiple studies and organisms giving us the ability to harness the power of a larger dataset than is typically feasible to obtain in a single lab. We then examined several machine learning algorithms to identify the best model for predicting age via highly conserved transcriptome signatures. Using this method, we analyzed publicly available RNA sequencing data from aging *Drosophila* and humans to reveal aging-associated genes with greater statistical power than can typically be obtained from a single laboratory. Our transcriptome profiling across species uncovered 50 genes whose expression is strongly correlated with aging in both *Drosophila* and humans. Twenty-two of these genes had not been previously associated with aging **(S4 Table)**.

Previous work supports a cross-species approach to the discovery of gene function in aging. Zullo et al. [43] demonstrated that longevity in humans is related to cortex transcriptome signatures, where genes underlying neural excitation and synaptic regulation are downregulated during aging. By comparing *C*. *elegans* with humans, they demonstrate that neural excitation increases as a function of age and inhibiting the excitation of neurons increases longevity. Other key mechanistic relationships such as NADH dehydrogenase expression and longevity [44] as well as dysfunction of mitochondrial proteins and aging [45] have been shown to be highly conserved between invertebrates and humans.

Important consideration must be taken when examining equivalence of invertebrate age to human age. There are several published reports comparing both mouse [46] and rat age [47, 48] to humans, each of which describe different methods for comparing chronological age across species. Although there is not a well-accepted formula for direct conversion between *Drosophila* age in days to human age in years [49], *Drosophila* is a common model of aging as denoted by De Nobrega and colleagues [50]. For our work, we created chronological age group cutoffs as indicated in methods based on a similar age comparison to previously published work [51].

### Predicted genetic pathways

Our analysis identified an enrichment of two established key aging-related pathways. The PI3K-Akt signaling pathway was heavily represented in our list of 50 genes with 10 being known players in this signaling pathway. This pathway has previously been shown to indirectly promote mTOR complex 1 and mTOR complex 2 kinases [52, 53] as well as decreased levels of Telomeric repeat-binding factor 1 (TRF1) [54]. TRF1 is part of a telomere protective complex which, if lost, results in increased telomere damage. Of the 10 genes found in the PI3K-Akt pathway, two were present in neurodegenerative disease designated datasets in STRING. ERBB2 has previously been shown to decrease expression in the hippocampus of normal adult mice and humans with increased expression in the hippocampus linked to Alzheimer’s disease [55]. Our study demonstrates an increase of ERBB2 expression in the PFC in normal aging, supporting further study into the role that this gene may be playing in the PFC as a risk factor in Alzheimer’s disease. Abnormal signaling in the PI3K-Akt pathway has been shown to lead to hyperphosphorylation of tau, one of the trademarks of Alzheimer’s disease [52]. One of the functions of the PI3K-Akt pathway is regulation of telomere health [53]. Telomere shortening has been demonstrated as a risk factor of various types of neurodegeneration [56] making ERBB2 an interesting target for further study.

Six of our 50 genes were found to be involved in the MAPK signaling pathway. The MAPK signaling cascade has known involvement in neurodegenerative diseases. Activation of MAPK has been shown to increase oxidative stress, which is a key risk factor in both Alzheimer’s and Parkinson’s diseases [57]. Our data also indicated ERBB2 involved in the MAPK signaling pathway. Proper MAPK signaling is crucial for maintaining homeostasis of cell proliferation and differentiation. Over-activation of MAPK signaling has been linked to neuronal inflammation, neuronal death, autophagy, and general Parkinson’s disease phenotypes [58]. Observing levels of ERBB2 with respect to the PI3k-Akt and MAPK signaling pathway could elucidate its role in neurodegenerative diseases. Mutations of ERBB2 could provide key answers into its role in Alzheimer’s and Parkinson’s diseases.

Given the strong correlation between aging and neurodegeneration, we were interested in identifying genes in our list of 50 highly conserved aging genes found in the human PFC that were known to be associated with neurodegenerative diseases. We referenced our list of 50 highly predictive aging genes with 1000 genes associated with Alzheimer’s and Parkinson’s diseases according to the STRING disease database. Given these genes reported associated in neuronal disease alongside conserved expression changes within aging we would propose further mechanistic studies of these genes as risk factors for neurodegenerative diseases associated with aging. These include Caspase-6 (CASP6) whose proteolytically processed form has been shown to increase in correlation with amyloid beta pathologies in Alzheimer’s disease patients [59]. We also identified DDIT4, a known inhibitor of the mTOR pathway whose specific role in aging has not yet been determined [60]. BMP4 has indicated its key role in neurogenesis of Alzheimer’s disease models through downregulation of neurogenesis in the dentate gyrus [61] but has not been studied in the PFC where our observed increased expression in aging may indicate its ability to play a compensatory role. Similarly, decreased expression of CSTB is correlated with disease where mRNA abundance in the peripheral blood has been shown to be decreased in patients with Parkinson’s disease [62]. DPM3 has also been shown to be downregulated in the entorhinal cortex of patients with Alzheimer’s disease [63]. However, all these genes require further study as to their functional role in the PFC.

In the current study, we focused on the human prefrontal cortex (PFC) and the *Drosophila* head due to the morphological changes in the PFC that often accompany neurological decline. However, comparison of tissue between human PFC and the *Drosophila* head should be exercised with caution. Numerous tissues outside of the brain exist within the *Drosophila* head [7].

Our data analysis validated 28 genes previously associated with aging that are conserved between human and *Drosophila*. Additionally, we identified 22 genes that do not have a previously characterized role in aging. This provides exciting insight into potential targets to follow-up utilizing the *Drosophila* model. The gonadotropin-hormone pathway is not well studied in *Drosophila*, yet our data suggests that a small number of conserved age related genes appear to be involved in this pathway in humans. Further investigation into these genes could provide more understanding for how the gonadotropin pathway and other pathways which have not been well studied in aging can be elucidated in the *Drosophila* model.

### Age prediction with machine learning

Other research groups have applied machine learning techniques to predict age from gene expression and biomarker data [64]. In humans, gene expression data from tissue culture of human dermal fibroblasts has been used to train an ensemble of linear discriminant analysis (LDA) classifiers achieving a median absolute error of 4 years and mean absolute error of 7.7 years [43]. We found that LDA performed much worse on our dataset than boosting algorithms (see **Table 2**). This may be due to the use of heterogeneous tissue samples from the human PFC containing several cell types (e.g., glia, neurons, astrocytes, oligodendrocytes) in the current study compared to tissue culture of a single cell type. Similarly, linear regression has been used to predict age from gene expression in peripheral blood samples [40] but performed poorly on our dataset. When sufficient training data is available, deep neural networks are an attractive option, as demonstrated in [29], where standard blood biomarkers were used to estimate age. With a sample size of 116 human PFC transcriptome profiles, deep learning approaches were not feasible in the current study. Instead, we sought to use approaches that require significantly less data to train. We found that the XGBoost algorithm created the best predictors of human age given human and *Drosophila* RNA sequencing data among 13 popular machine learning algorithms, as shown in **Table 2**. We selected the XGBoost algorithm because it performed well in age prediction with human and fly data; however, several other machine learning algorithms could generate models with very high accuracy and low error, especially in fly.

Due to the nature of data collection in animal experiments, samples are normally collected at a few specific ages, such as 10 days or 25 days. The variability in gene expression data from a single time point is likely much less than the variability across a range of ages (e.g., 5–20 days). As a result, models trained on data from a few time points might generalize poorly to new samples from time points never seen by the algorithm. This may explain some of the performance gap between aging via fluorometrically measured pteridine 6-biopterin [65] and our approach. This limitation of the data did not occur with human samples which display a more continuous span of ages. As is common with the use of secondary data there was some imbalance in the number of samples between age groups in fly (young: 30, middle: 24, old: 54); however, **S2 Fig** indicates that misclassification rates were similar in the three age groups.

Reproducibility and transparency are important current issues in the field of machine learning [66], where models using different random seeds may produce widely different results from one another, ultimately producing data that look as if they come from completely different distributions [67]. To combat this issue, our models were run across 1000 random permutations of data, and we reported averages and confidence intervals for each result. Additionally, genetic data is highly correlated in nature, which may explain why several classification models incorrectly identified some subjects across several re-samplings, highlighting XGBoost’s resilience to bias from multicollinearity. While multicollinearity may explain part of these results, it is unclear if subjects that were incorrectly classified across multiple random draws displayed inherently different transcriptome expression profiles than other subjects at a similar chronological age, or if these errors were due to the inherent random nature of the model. Future studies examining the predictive utility of neurological transcriptome data should work toward determining why specific samples in an age category display different transcriptome signatures, if any arise.

Several problems are inherent to combining data from publicly available repositories. Performing meta-analysis across multiple RNA sequencing datasets is limited by the lack of normalization standards [68] and limited documentation requirements for submitting data to these repositories. Numerous other studies posted on the NIH SRA contained potentially appropriate data to include in our study but did not include any descriptive metadata, preventing us from evaluating whether inclusion or exclusion criteria were met. Enhancing the available data descriptors for publicly available data would enable widespread use of these sequencing data and remove the systematic bias due to the lack of documentation [68]. Another source of bias in meta-analysis of public data is bias towards males. Nearly all the publicly available samples were male, resulting in less than 10% of the samples used in this analysis being from females. The large, unbalanced distribution of biological sexes may have skewed the data to overrepresent age-related changes occurring primarily in males. This limitation could be addressed by future studies including more samples of both biological sexes and making all samples publicly available.

A key strength of this study was that we achieved a large sample size by combining RNA seq samples across studies to improve the overall statistical power. Additionally, we were able to overcome challenges of using gene expression data with different underlying read count distributions due to differences in sample depth across studies through normalizing read counts using the TMM method. Differences among individual datasets still exist, illustrating the necessity for new statistical approaches to combine RNA sequencing data more effectively across multiple studies generated by different sequencers, RNA extraction protocols, library preparation kits, and experimental design of sequencing.

In this study, we uncovered 50 genes conserved between humans and flies capable of accurately predicting age in both species. Roughly half of these genes have been previously associated with aging. We demonstrate that of our 50 genes, 22 novel genes have not yet been published with respect to aging (see **S5 Table**). Further study of the novel 22 aging-associated genes may shed light on previously unassociated pathways that are altered during aging processes in the PFC. These results position scientists to delve deeper into the underlying conserved mechanisms of these identified aging genes in the PFC. Manipulating the expression of these conserved aging genes could potentially extend lifespan or enable development of novel anti-aging therapeutic compounds. Our dataset combining publicly available transcriptome data across multiple studies and two species demonstrated successful identification of chronological age using neurological transcriptome signatures. Using machine learning to model aging, we identified novel similarities in aging signatures across humans and *Drosophila* emphasizing the necessity for additional comparative aging research studies.

## Supporting information

S1 Table Aging correlated genes. This table depicts the aging correlated genes for humans and flies sorted according to their correlation coefficient.
(XLSX)

S2 Table Regression tables predicting chronological age. This table depicts the average R^2^, mean square error, median absolute error and R^2^ 95% confidence interval across 1000 iterations of training/testing predictions. Each row represents a different way to select genetic features for age prediction, where each column represents the metric used for evaluating the effectiveness in predicting aging.
(XLSX)

S3 Table Classification tables for age group prediction. This table depicts the average accuracy, F1, Precision, and Recall scores for classifying samples into their age groups. The data contained in these tables represents average scores across 1000 iterations of training/testing predictions. Each row represents a different way to select genetic features for age prediction, where each column represents the metric used for evaluating the effectiveness in age group classification.
(XLSX)

S4 Table Top 50 conserved aging predictive genes. This table describes whether previous reports exist linking these genes to aging or neurodegeneration phenotypes in Human or another model organism.
(XLSX)

S5 Table Novel 22 conserved aging predictive genes. This table describes previous literature of listed genes, along with references.
(XLSX)

S1 Fig Normalization analysis. Histograms of log_2_ of read counts by study indicate improved distribution overlap following normalization. A) Results without normalization applied. B) Results after applying Relative Log Expression (RLE) normalization. C) Results after applying Trimmed Mean of M values (TMM) normalization. Each color corresponds to a different study.
(TIF)

S2 Fig Biological age prediction using XGBoost across species in classification. Confusion matrices of average biological age prediction. A) Average age group classification results for human samples using all available data. B) Average age group classification results for Drosophila samples using all available data. C) Average age group classification results for 1000 genes in human most correlated with aging. D) Average age group classification results for 1000 genes more correlated with aging in Drosophila. E) Average age group classification results for 50 conserved and correlated genes applied to predict age in humans. F) Average age group classification results for 50 conserved and correlated genes applied to predict age in Drosophila. All confusion matrixes depict the average of 1000 trials of age prediction.
(TIF)

S3 Fig Panther pathway analysis and phylogenetic relationship. Panther pathway analysis of genes implicated in aging. A) Predicted pathways using 1000 genes most associated with aging in human data. Threshold set at 5 genes involved in a pathway. B) Pathway analysis of 50 human genes conserved in fly. C) Phylogenetic tree branch depicting gene sequence homology. Genes included are found in the Gonadotropin-releasing hormone pathway (P06664). Numbers throughout the branches indicate bootstrapped scores out of 100 trails testing for sequence similarity. Higher numbers indicate stronger prediction of phylogenetic relationship.
(TIF)
