# Supplementary figures and images for: Identification of conserved transcriptome features between humans and Drosophila in the aging brain utilizing machine learning on combined data from the NIH Sequence Read Archive
Source: PLoS One. 2021 Aug 11;16(8):e0255085. doi: 10.1371/journal.pone.0255085 (PMC8357136; doi:10.1371/journal.pone.0255085)

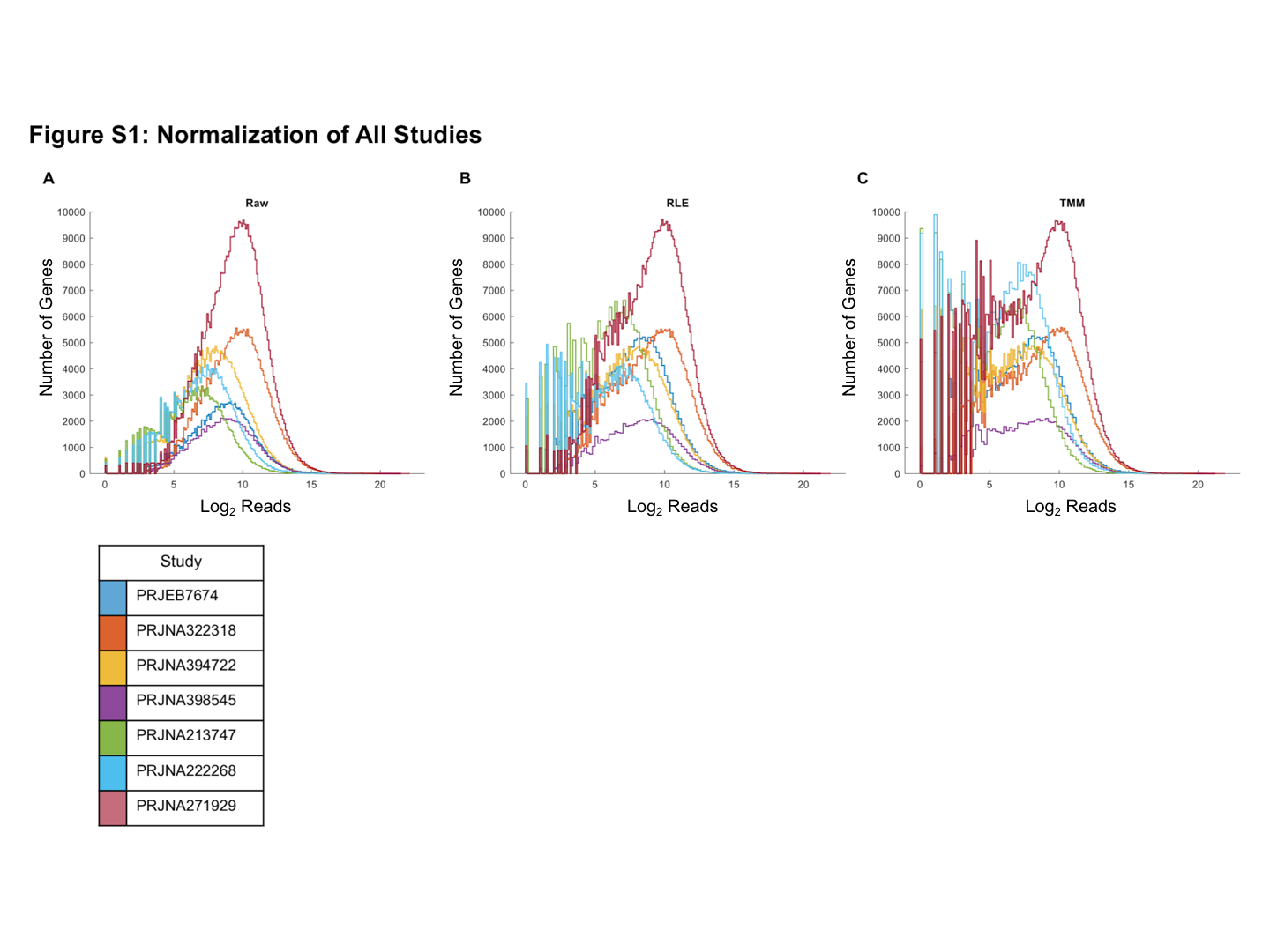

Supplement: S1 Fig — Histograms of log2 of read counts by study indicate improved distribution overlap following normalization. A) Results without normalization applied. B) Results after applying Relative Log Expression (RLE) normalization. C) Results after applying Trimmed Mean of M values (TMM) normalization. Each color corresponds to a different study. (TIF) [file pone.0255085.s006.tif]

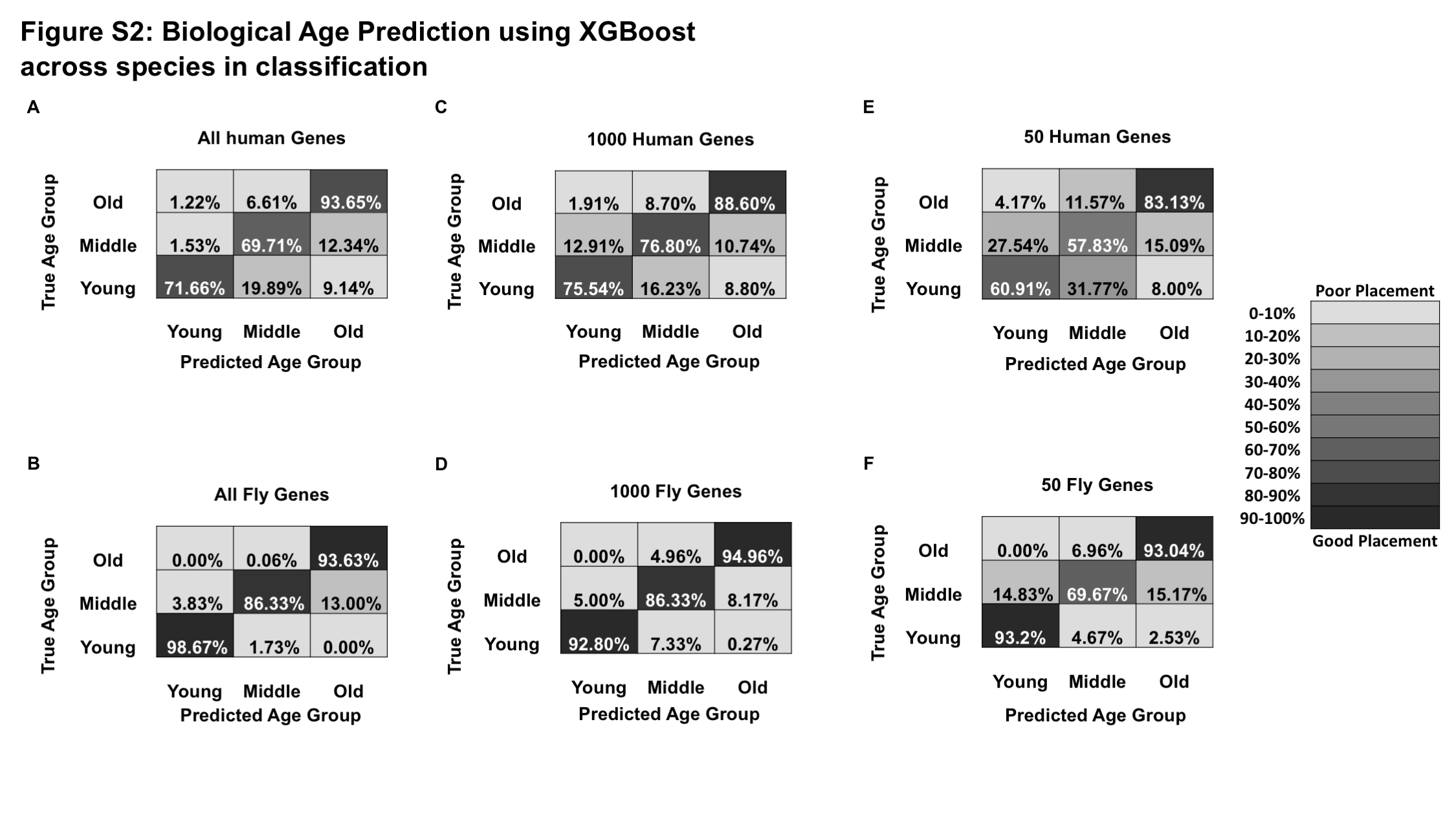

Supplement: S2 Fig — Confusion matrices of average biological age prediction. A) Average age group classification results for human samples using all available data. B) Average age group classification results for Drosophila samples using all available data. C) Average age group classification results for 1000 genes in human most correlated with aging. D) Average age group classification results for 1000 genes more correlated with aging in Drosophila. E) Average age group classification results for 50 conserved and correlated genes applied to predict age in humans. F) Average age group classification results for 50 conserved and correlated genes applied to predict age in Drosophila. All confusion matrixes depict the average of 1000 trials of age prediction. (TIF) [file pone.0255085.s007.tif]

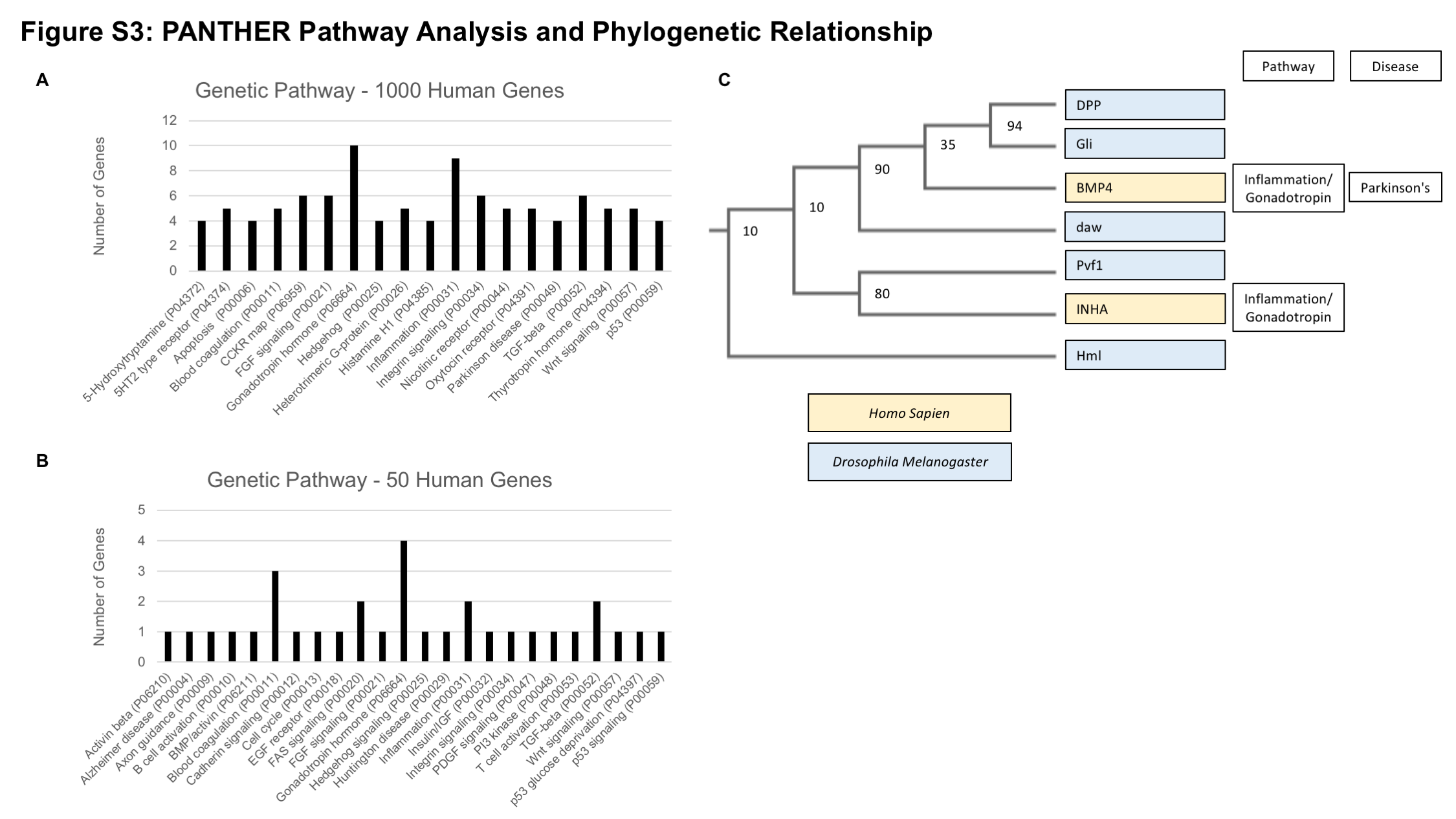

Supplement: S3 Fig — Panther pathway analysis of genes implicated in aging. A) Predicted pathways using 1000 genes most associated with aging in human data. Threshold set at 5 genes involved in a pathway. B) Pathway analysis of 50 human genes conserved in fly. C) Phylogenetic tree branch depicting gene sequence homology. Genes included are found in the Gonadotropin-releasing hormone pathway (P06664). Numbers throughout the branches indicate bootstrapped scores out of 100 trails testing for sequence similarity. Higher numbers indicate stronger prediction of phylogenetic relationship. (TIF) [file pone.0255085.s008.tif]
